# Ligands in crystal structures that aid in functional characterization

**DOI:** 10.1107/S1744309110035748

**Published:** 2010-09-30

**Authors:** Anna E. Speers, Benjamin F. Cravatt

**Affiliations:** a The Department of Chemical Physiology and The Skaggs Institute for Chemical Biology, The Scripps Research Institute, 10550 North Torrey Pines Road, La Jolla, California, USA

**Keywords:** ligands, structural genomics

## Abstract

Liganded structures can be instrumental in assigning function to uncharacterized proteins by revealing active sites, conserved residues, binding motifs, and substrate specificity. This introduction provides an overview and commentary on the value of liganded structures emerging from the JCSG structural genomics initiative.

## Abstract 2

An overview and commentary on the value of liganded structures emerging from the JCSG structural genomics initiative.

One of the primary goals of proteomics research is to assign functional roles to uncharacterized proteins, which are estimated to comprise at least 40% of human and other genomes (Pawlowski, 2008 ▶). Towards this goal, structural genomics efforts have provided invaluable insights in the form of protein structures with functionally relevant ligands. These ligands may be endogenous small molecules (*e.g.* metal ions, cofactors, substrates, products and inhibitors) that are incorporated during protein expression, or exogenous agents (*e.g.* metal ions, buffer components, salts, precipitants and cryogens) that are acquired during purification and/or crystallization. While many of the observed ligands fall into the former category, non-native ligands can act as analogs of endogenous species, providing functional insights akin to those of native ligands.

Liganded structures can be instrumental in assigning function to uncharacterized proteins by revealing active sites, conserved residues, binding motifs and substrate specificity. Moreover, these ligand-based structural insights can help to classify proteins of unknown function into subgroups and/or within known classes based on conserved features that may not be evident from primary sequence analysis alone. The Protein Structure Initiative (PSI; http://www.nigms.nih.gov/Initiatives/PSI/) is a significant repository of liganded structures, with the majority (65%) of their 4000+ entries containing bound ligands. Importantly, the Joint Center for Structural Genomics (JCSG; http://www.jcsg.org) has now developed a publically available search tool, the Ligand Search Server (http://smb.slac.stanford.edu/jcsg/Ligand_Search/), to mine the entire database of PSI structures for these bound ligands, enabling functional insight into these uncharacterized species (Kumar *et al.*, 2010 ▶). Interestingly, of the ∼600 PSI structures identified by the server as proteins lacking functional annotation, about two-thirds have one or more bound ligands which could potentially be used to infer function. For details on how ligands are identified at the JCSG in the course of structure determination, see Kumar *et al.* (2010 ▶). For example, Kumar *et al.* (2010 ▶) highlight how a bound metal ion (in this case zinc) was instrumental in locating the active sites of three proteins of unknown function: YP_164873.1 (PDB code 3chv; JCSG, unpublished work), YP_555544.1 (PDB code 3e02; JCSG, unpublished work) and YP_556190.1 (PDB code 3e49; JCSG, unpublished work). Moreover, these proteins were observed to bind zinc in a manner similar to the known 3-keto-5-amino­hexanoate cleavage protein (YP_293392.1 from *Ralstonia eutropha* Jmp123; PDB code 3c6c; JCSG, unpublished work), allowing functional classification (Fig. 1 ▶). Because the sequence identities for all four proteins are low (∼30%), it is unlikely that this homology would have been identified from sequence alignment alone.

Kumar *et al.* (2010 ▶) also discuss several examples of how common reagents can mimic biological molecules: in the case of YP_001181608.1 (PDB code 3gxg; JCSG, unpublished work), a sulfate ion mimics a phosphate ion, suggesting phosphatase activity; for YP_001089791.1 (PDB code 3g68; JCSG, unpublished work), a bound citrate allowed localization of the active site; and a Tris molecule found in the active site of YP_001304206.1 (PDB code 3h3l; JCSG, unpublished work) mimicked the binding of a sugar moiety (see Figs. 4*a* and 5 of Kumar *et al.*, 2010 ▶). Even unknown ligands (UNLs) can provide mechanistic insights, such as in the uncharacterized protein NP_823353.1 (PDB code 3giw; JCSG, unpublished work), in which the UNL resembles phenylalanine (Fig. 2 ▶). This information, together with an overall structural similarity to a specific class of methyltransferases, suggested a possible catalytic role for this enzyme (Kumar *et al.*, 2010 ▶).

Overall, the JCSG has been the largest contributor of ligand-containing structures to the PSI database, including over 100 structures containing unknown ligands and several structures with ligands that are unique in the PDB (Kumar *et al.*, 2010 ▶). The JCSG has a long history of focusing on the functional significance of ligand-containing structures. The first protein structure to be determined by the JCSG, the thymidylate synthase Thy1 from *Thermotoga maritima*, revealed a novel fold and contained complexed FAD molecules at each of the active sites of the tetramer, suggesting a putative mechanism that distinguishes the enzyme from other thymidylate synthases (Koehn *et al.*, 2009 ▶). More recently, the JCSG published a crystal structure (PDB code 3bos) of the protein Hda (homologous to DnaA), an essential component of the complex responsible for the regulatory inactivation of DnaA during chromosomal replication, in which the authors identified a bound CDP and inferred that it initiates and maintains Hda in an inactive conformation which may serve an important regulatory role (Xu, McMullan *et al.*, 2009 ▶).

In some cases, structures with bound ligands can help to classify entire classes of proteins by providing key insights into the conserved residues important for cofactor/substrate binding. One such example of profound structural insights from the JCSG is the NlpC/P60 family of cysteine peptidases, which are involved in cell-wall recycling. The JCSG recently solved the structure of one such protein, YkfC from *Bacillus cereus* (NP_979181.1; PDB code 3h41), in complex with an endogenously derived bound reaction product, l-Ala-γ-d-Glu (Xu *et al.*, 2010 ▶). Previously, the JCSG had determined the crystal structure of another NlpC/P60 enzyme, *Av*PCP from *Anabaena variabilis*, which contained a region of disordered electron density, half of which could be discerned as l-alanine (Xu, Sudek *et al.*, 2009 ▶). The location and orientation within the active site, along with molecular-docking studies, suggested a highly tailored substrate specificity for short cell-wall-derived peptides with an N-terminal l-alanine. In the case of *Bc*YkfC, the presence of the more structurally defined dipeptide allowed identification of the key residues and domains involved in substrate specificity, which in turn provided a basis for the previously observed substrate specificity of NlpC/P60-family members and allowed the classification of a large subfamily of highly specialized NlpC/P60 enzymes, many of which previously escaped classification based on whole-protein sequence homology (Xu *et al.*, 2010 ▶).

Liganded structures from the JCSG also allowed the classification of a novel family of peroxidases. Peroxidases are employed by all living organisms for heme-dependent oxidations in biosynthesis, degradation, defense and oxidative-stress responses. Recently, a new family of dye-decolorizing peroxidases (DyPs) has been identified. The DyP enzymes show little sequence and structural homology to existing peroxidases and lack several conserved residues. Previous biochemical and modeling studies of DyP-family members had returned conflicting evidence regarding the location of the heme-binding site, its occupancy in oligomeric structures and the key residues involved in heme stabilization. The JCSG published the first structure of a DyP peroxidase, TyrA from *Thanatephorus cucumeris* (NP_810132.1; PDB code 2gvk; Zubieta, Krishna *et al.*, 2007 ▶). Structural analysis and biophysical characterization of this protein indicated that it bound heme (Zubieta, Krishna *et al.*, 2007 ▶). Subsequent structures of complexes with heme (PDB code 2d3q, T. Sato, Y. Sugano & M. Shoda, unpublished work; PDB code 2iiz (Zubieta, Joseph *et al.*, 2007 ▶) allowed the first unambiguous characterization of the active site, including the basis of the observed low-pH activity and the relationship between heme binding and oligomerization (Zubieta, Joseph *et al.*, 2007 ▶). Identification of the active site from this liganded structure also allowed analysis of substrate specificity, which appears to be tailored to small hydrophobic molecules and is consistent with the putative role of TyrA in melanin biosynthesis. Identification of the conserved residues, which maintain the heme in a novel ‘bent’ conformation, greatly facilitated the classification of a multitude of other DyP-family members.

In this issue of *Acta Crystallographica Section F*, several recent instances of liganded protein structures that provide key insights into protein function are discussed. One manuscript (Han *et al.*, 2010 ▶) details the first crystal structure of a tryptophanyl tRNA-synthetase (TrpRS) containing a [4Fe–4S] iron–sulfur cluster. The cluster-binding motif is conserved throughout anaerobic proteobacteria and archaea; however, this TrpRS structure from *Thermotoga maritima* is the first structure to be solved with the bound cluster. Interestingly, the presence of the cluster/binding motif is unique to TrpRS among the 20 tRNA synthetases. Based on the specific location of the iron–sulfur cluster and a similar cluster alignment found in the enzyme responsible for tRNA modification, the authors suggest that a putative role for the iron–sulfur cluster is in the recognization of post-transcriptionally modified tRNA, which has been linked to the enhanced fidelity of mRNA translation in response to environmental and other stress factors.

A second manuscript details the first structures of three tungsten formylmethanofuran dehydrogenase subunit E (FwdE) proteins, which are believed to play a role in microbial methane production (Axelrod, Das *et al.*, 2010 ▶). FwdE structures from three different microorganisms, DSY1837 (*Desulfitobacterium hafniense*; PDB code 2glz), Ta1109 (*Thermoplasma acidophilum*; PDB code 2gvi) and SYN_00638 (*Syntrophus aciditrophicus* Sb; PDB code 3d00), were determined, each with a unique metal-binding architecture. All three proteins contain an N-terminal α+β core domain (NTD) and two of the three (TA1109 and SYN_00638) also contain a C-terminal zinc-finger domain which interacts with the respective NTD near the putative active site. Despite the overall structural similarity of the NTDs (overlap analysis: r.m.s.d. overlap of 1.9 Å for 128 C^α^ atoms for DSY and Ta, r.m.s.d. overlap of 2.6 Å for 122 C^α^ atoms for DSY and SYN, r.m.s.d. overlap of 2.6 Å for 114 C^α^ atoms for Ta and SYN), the three domains exhibit dissimilar metal-binding properties. The structure of DSY1837 revealed a tetrahedral metal-binding pocket with mixed Zn/Ni cation occupancy, whereas the analogous binding site for TA1109 contained only Zn and that of SYN_00638 contained only two of the four metal-binding residues and no bound cation; however, a Cl anion was observed to bind in an adjacent region of the SYN_00638 NTD domain. The presence of such a diversity of cation/anion-binding architectures near the purported active sites suggests varied substrate/effector specificities for these enzymes.

Also reported in this issue is the crystal structure of XcTcmJ, a *Xanthomonas campestris* pv. *campestris* cupin protein of unknown function, which was recently crystallized with a bound metal ion (Axelrod, Kozbial *et al.*, 2010 ▶). The cupin superfamily is subdivided into more than 18 functional classes, with catalytic diversity believed to be influenced by the presence/identity of the bound metal cofactor, which is observed in the crystal structures of most cupins. However, a previously reported structure for the apo form of XcTcmJ did not contain a bound metal ion (Chin *et al.*, 2006 ▶), bringing into question the metal-dependency of this enzyme. In the present report, Axelrod and coworkers detail a crystal structure of XcTcmJ with zinc acetate in the putative active site and with a significant reorganization of a conserved histidine ligand and several nearby residues to accommodate the putative cofactor, indicating a functional role for zinc. The binding-site architecture supports an octahedral coordination for zinc, which, in conjunction with the presence of a coordinating water molecule, suggests a catalytic (rather than a structural) role for zinc and conclusively answers the question of the metal dependence of XcTcmJ.

As these examples demonstrate, protein structures with ligand inclusions can have profound functional implications. Importantly, non-native as well as native ligands can provide important insights. This issue of *Acta Crystallographica Section F* highlights the value of liganded structures emerging from structural genomics endeavors. Looking forward, the JCSG’s Ligand Search Server will enable more complete mining of ligand-bound protein structures, greatly facilitating the characterization of unknown proteins.

## Figures and Tables

**Figure 1:**
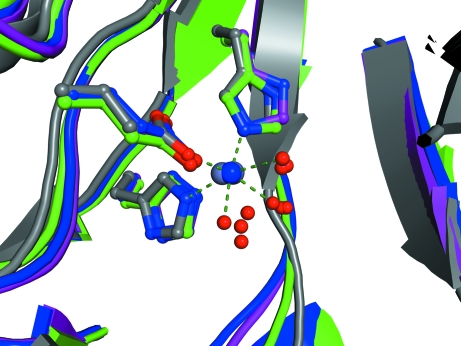
Metal ligands were instrumental in locating the active sites of three uncharacterized proteins. Comparison of the metal-bound crystal structures of YP_164873.1 (blue), YP_555544.1 (green) and YP_556190.1 (magenta) with that of the known 3-keto-5-aminohexanoate cleavage protein (grey) reveals a similar metal-binding motif, allowing unambiguous identification of the enzyme active sites and functional classification. Hydrogen bonds are shown as dashes, water molecules as red spheres, Zn atoms as grey spheres and Ni as a blue sphere. The protein is rendered with cartoon-representation side chains and the active-site His and Asp residues are shown in ball-and-stick representation.

**Figure 2:**
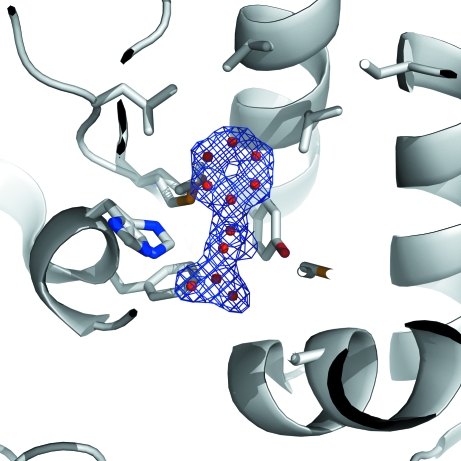
Crystal structure of the uncharacterized protein NP_823353.1 with a bound UNL resembling phenylalanine. UNL (unidentified ligand) atoms are represented as red spheres enveloped by the electron-density mesh (2*F*_o_ − *F*_c_ density contoured at 1σ level above the mean) and surrounded by the protein rendered in cartoon representation. Although the ligand looks like phenylalanine, it was annotated as a UNL since no definitive proof of its identity by other methods was available.
